# Molecular Mapping and CRISPR/Cas9 Validation Reveal That *SlVQ10* Regulates Seed Size in Tomato

**DOI:** 10.3390/plants15152363

**Published:** 2026-07-31

**Authors:** Tianlin Sun, Liwei Chen, Xiuqing Zhu, Tong Pei, Yue Wang, Chunxin Liu, He Zhang, Dalong Li, Tingting Zhao, Xiangyang Xu

**Affiliations:** 1 College of Horticulture, Northeast Agricultural University, Harbin 150030, China; 15633675026@163.com (T.S.); 15178383537@163.com (L.C.); 13231182160@163.com (X.Z.); pt_95624@163.com (T.P.); yuewang20241015@163.com (Y.W.); llucky992025@163.com (C.L.); esculentum@neau.edu.cn (H.Z.); lidalong@neau.edu.cn (D.L.); 2 Key Laboratory of Biology and Genetic Improvement of Horticultural Crops (Northeast Region), Ministry of Agriculture and Rural Affairs, Northeast Agricultural University, Harbin 150030, China

**Keywords:** *Solanum lycopersicum*, seed size, VQ protein, EMS mutant, BSA–seq, KASP, CRISPR/Cas9, fine mapping

## Abstract

Seed size affects seed vigor, seedling establishment, and seed utilization in tomato. Yet the genetic basis of this trait remains poorly defined in tomato itself. Here, we describe a stable small–seed mutant, T31, isolated from an ethyl methanesulfonate (EMS)–mutagenized population of the elite inbred line DL5. Relative to the wild type, T31 showed a 24% reduction in seed width, whereas vegetative growth and major fruit traits were largely unchanged. Throughout this study, ‘seed size’ refers to seed width, which was used as the principal index of seed size because tomato seeds are oblate. Genetic analysis of six populations (P_1_, P_2_, F_1_, F_2_, BC_1_, and BC_2_) indicated that the phenotype is controlled by a single recessive locus, designated *ssm*. Bulked segregant analysis sequencing (BSA–seq) placed *ssm* within a 1.77 –Mb interval on chromosome 4. KASP–based fine mapping reduced this interval to 390 kb and identified six EMS–type SNPs. Only one of these SNPs was located in an exon of *Solyc04g073950.2*, where it caused a Pro337Ser substitution. This gene encodes a VQ motif–containing protein and was designated *SlVQ10*. To test gene function, we generated CRISPR/Cas9 knockout lines in the DL5 background. Two independent homozygous knockout lines reproduced the small–seed phenotype. Seed size was reduced by 31–33%, and thousand–seed weight decreased by 33–35%. Histological analysis further showed reduced seed–coat cell expansion in the mutants. Together, the genetic and genome–editing data support *SlVQ10* as the gene underlying *ssm* and indicate that it promotes seed size in tomato.

## 1. Introduction

Seed size is a fundamental agronomic trait. It influences reserve accumulation, seed vigor, seedling establishment, and early crop performance. It also affects how seed quality is judged in breeding and seed production. In tomato, however, seed traits have usually received less attention than fruit traits. As a result, the genetic basis of seed–size variation remains much less clear than the basis of fruit size, fruit shape, or ripening.

Recent research has clarified the molecular basis of seed–size control. Maternal pathways, endosperm–derived signals, and embryo growth do not act separately. They form a connected network. Across species, several recurring processes have been identified. These include hormone signaling, ubiquitin–dependent regulation, transcriptional control, and the control of cell proliferation and cell expansion. Early endosperm development is now a central theme in this field. The length of the coenocytic stage, the timing of cellularization, and the physical properties of surrounding tissues can all affect final seed size. Maternal tissues are also active in this process. They provide signals rather than serving only as a structural boundary. Recent studies have reinforced this view and placed seed–coat–derived signals within the core regulatory framework of seed growth.

Seed size is therefore not set by a single tissue. It emerges from coordinated growth of the embryo, endosperm, and maternal seed–coat. Hormonal and mechanical constraints also matter. Maternal tissues are especially important because they shape both the growth environment and the signaling context of the developing seed.

Research in *Arabidopsis* and several crops has identified many pathways that influence seed size. These include the HAIKU pathway, hormone–related regulators, and genes that affect endosperm proliferation or cellularization [1,2,3,4,5,6,7,8]. However, the regulatory pathways controlling seed development in tomato remain far less resolved. Most studies in tomato have approached seed development through transgenes, stress responses, or general reproductive phenotypes rather than by cloning causal loci from stable mutants [9,10].

At the same time, tomato–specific molecular resources have improved. Stage–resolved transcriptome studies have revealed marked shifts in hormone–related genes, transcription factors, and starch–associated pathways during seed growth and maturation. Broader tissue atlases have also recovered seed–enriched expression patterns and developmental transitions, which can facilitate gene prioritization as well as functional analyses [11,12,13]. These advances make tomato a useful system for seed biology. It combines strong genomic resources, efficient transformation, and growing transcriptomic support. Tomato also offers a practical bridge between basic developmental genetics and trait analysis in a crop species. Even so, the field still needs more studies that move from mutant phenotype to causal gene and then to functional validation.

VQ motif–containing proteins are plant–specific regulators defined by a conserved VQ motif. Many act through WRKY transcription factors or MAPK–related pathways. They have been linked to growth, defense, and transcriptional control in several species [14,15,16,17]. In *Arabidopsis*, IKU1 provides direct evidence that a VQ protein can regulate seed size through effects on endosperm development [2]. In rice, *OsVQ13* overexpression increased grain length, width, and weight, providing direct evidence that a VQ protein can affect final grain size [18]. In maize, tea plant, and Triticeae genomes including barley, family–level analyses have revealed lineage–specific expansion and diverse expression patterns of VQ genes, suggesting that these proteins have been repeatedly recruited into developmental as well as stress–related pathways [19,20,21]. In poplar, VQ1 has been shown to participate in hormone–related signal transduction and the regulation of growth–related responses, supporting the view that VQ proteins are not limited to a single regulatory pathway [22]. Even so, direct evidence linking VQ proteins to seed–size control remains insufficient. In tomato, the VQ family has been described at the genome–wide level, but developmental evidence remains limited [17,23,24].

In this study, we identified a stable EMS–induced tomato small–seed mutant and used classical genetics, BSA–seq, KASP fine mapping, and CRISPR/Cas9 validation to resolve the underlying locus. These results provide strong genetic evidence for the causal locus and, together with the knockout analysis, identify *Solyc04g073950.2* as the gene responsible for the phenotype. The encoded protein belongs to the VQ motif–containing family, and we therefore designated it *SlVQ10*. This study provides direct evidence that a VQ protein regulates seed size in tomato.

## 2. Results

### 2.1. Phenotypic Characterization and Inheritance of the Small–Seed Mutant

**Figure 1:**
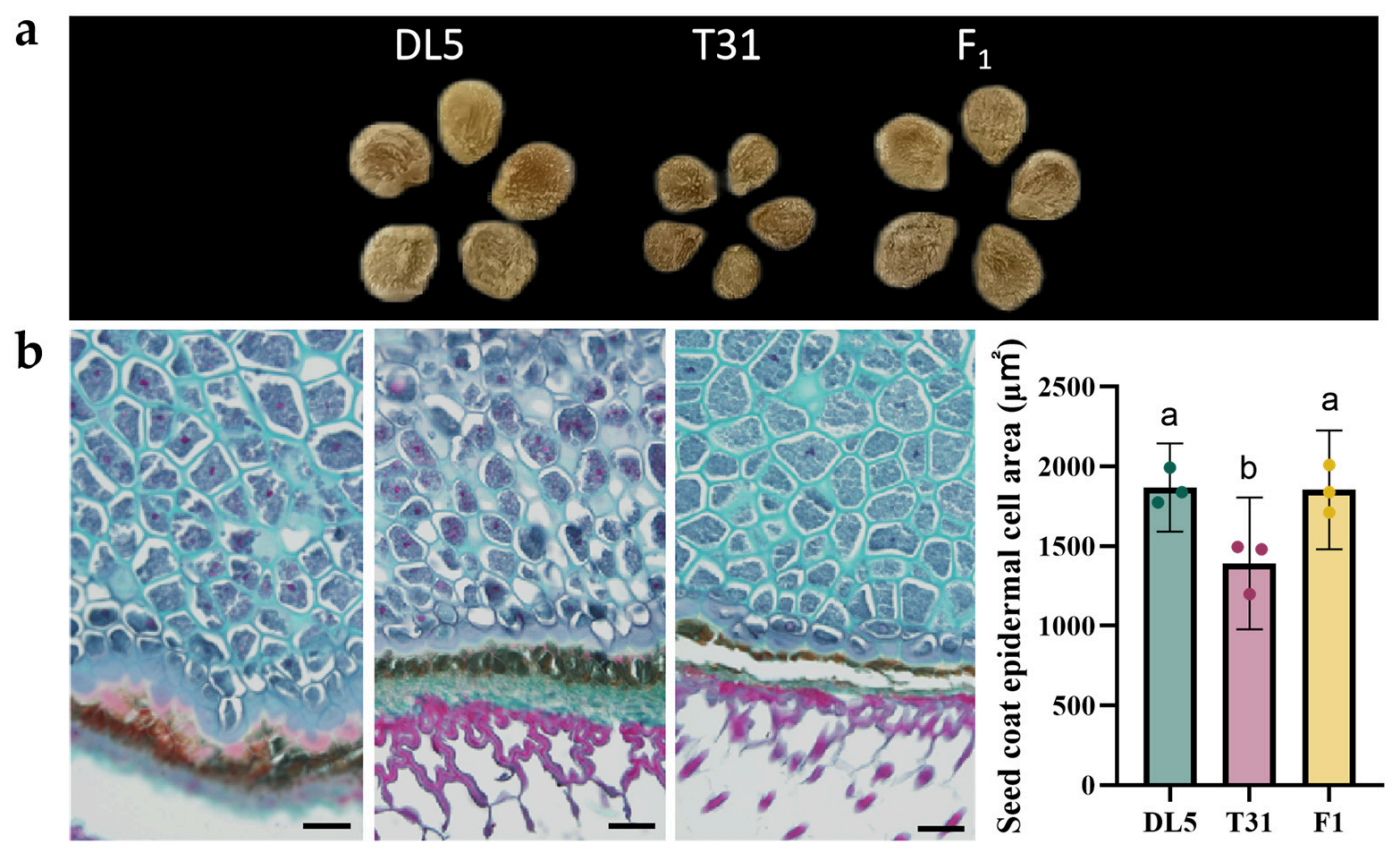
Seed morphology and cellular structure of DL5, T31, and F_1_ plants. (**a**) Representative seeds showing reduced size in T31 compared with DL5 and F_1_, while seed shape remains similar. (**b**) Paraffin sections showing smaller seed–coat cells in T31 than in DL5 and F_1_. Scale bars = 100 μm; seed–coat epidermal cell areas of different lines, measured from paraffin sections using Fiji (Fiji is just ImageJ)–win64. Data are presented as mean ± standard deviation (*n* = 3); Different letters indicate significant dif–ferences among all experimental conditions. Statistical significance was assessed by one–way ANOVA with Tukey’s multiple comparison test (*p* < 0.05).

Seeds of T31 were visibly smaller than those of DL5, while overall seed shape re–mained similar (Figure 1a). Histological sections further showed that seed–coat cells in T31 were smaller than those in DL5 and F_1_ plants. Quantification of seed–coat epidermal cell area confirmed this observation: mean cell area was 1868.33 ± 111.63 μm^2^ in DL5, 1391.00 ± 166.42 μm^2^ in T31 (a 25.6% reduction relative to DL5), and 1853.33 ± 150.01 μm^2^ in F_1_ (Figure 1b). One–way ANOVA followed by Tukey’s multiple comparison test showed that T31 differed significantly from both DL5 and F_1_ (*p* < 0.05), whereas DL5 and F_1_ did not differ significantly, indicating that reduced seed–coat cell expansion contributes to the mutant phenotype and segregates with the recessive small–seed trait.

**Figure 2:**
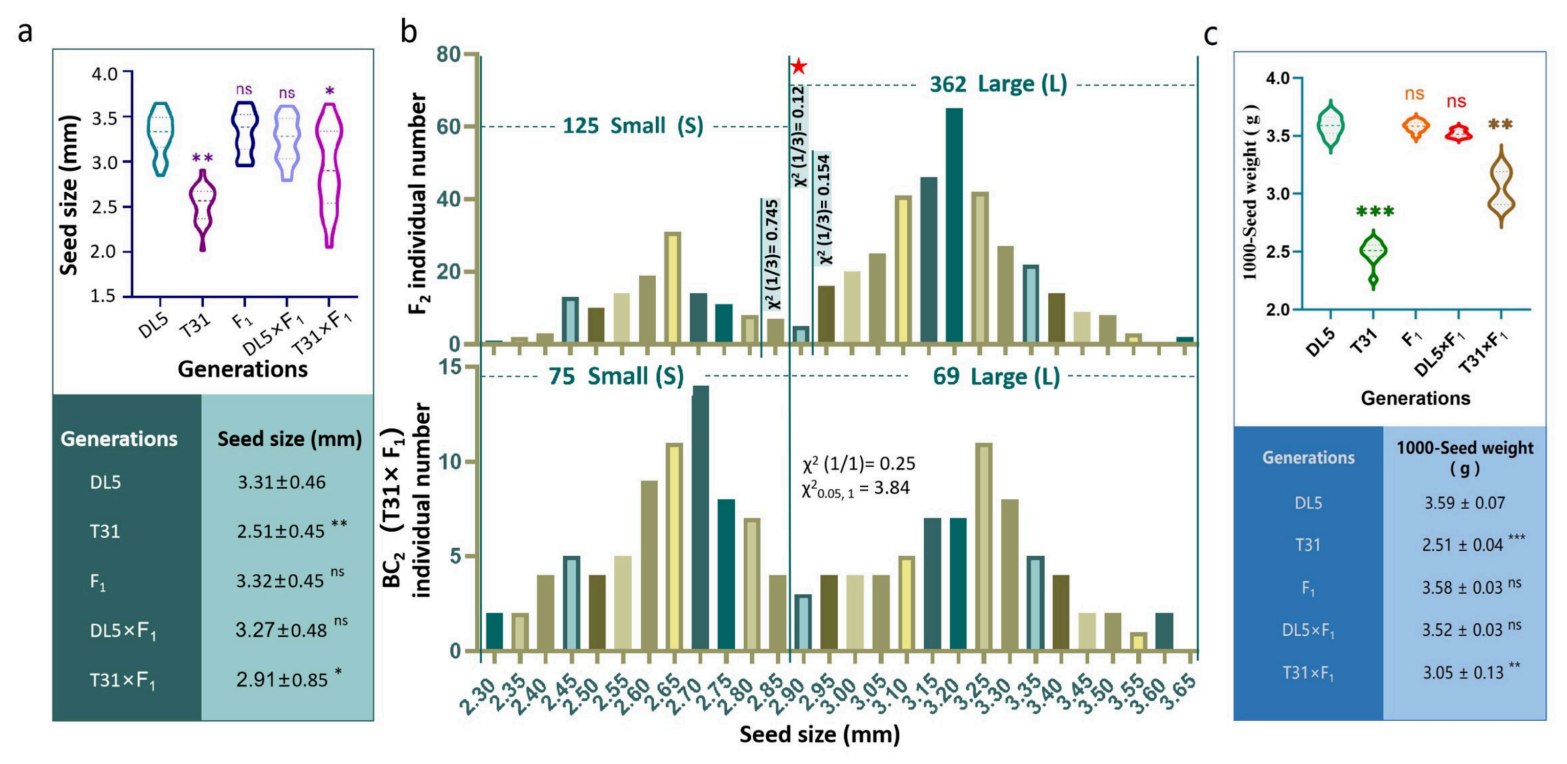
Seed size statistics and genetic analysis. (**a**) Seed size across six generations showing a significant reduction in T31 relative to DL5; F_1_ and BC_1_ resemble DL5. (**b**) Frequency distribution of seed size in the F_2_ population (*n* = 487) showing a bimodal pattern; Vertical lines mark the three candidate classification thresholds tested; the threshold ultimately adopted (2.85 mm, red star) gave the best fit to the expected 3:1 ratio (χ^2^ = 0.12) among the three candidates (χ^2^ = 0.745 and 0.154 for the alternative thresholds). (**c**) Thousand–seed weight across generations. Data are presented as mean ± standard deviation (*n* = 3); ns, not significant; *, *p* < 0.05; **, *p* < 0.01; ***, *p* < 0.001; Statistical significance was assessed by two–tailed Student’s *t*–test (*p* < 0.05).

Quantitative analysis showed that mean seed size was 3.31 ± 0.46 mm in DL5 and 2.51 ± 0.45 mm in T31, representing a 24% reduction in the mutant (*p* < 0.01; Figure 2a). Thousand–seed weight also differed markedly between the two lines, with values of 3.59 ± 0.07 g in DL5 and 2.51 ± 0.04 g in T31 (*p* < 0.0001; Figure 2c). In contrast, F_1_ and BC_1_ plants showed seed size and TSW values similar to those of DL5. Note that the numerical value for T31 thousand–seed weight (2.51 ± 0.04 g) coincidentally matches the value for T31 seed size (2.51 ± 0.45 mm); these are independent measurements of different traits.

The seed–size distribution in the F_2_ population was clearly bimodal (Figure 2b). The F_2_ seed size distribution showed three local minima (2.80, 2.85, and 2.90 mm) that could plausibly separate the two modes. We evaluated the goodness of fit to the expected 3:1 ratio at each candidate threshold (χ^2^ = 0.745, 0.12, and 0.154, respectively) and selected 2.85 mm, which gave the best fit to the expected segregation ratio, as the classification threshold. Using this threshold, the segregation of large–seed and small–seed plants fit a 3:1 ratio (χ^2^ = 0.12, *p* > 0.05). The BC_2_ population fit an approximate 1:1 segregation ratio (χ^2^ = 0.25, *p* > 0.05). These results indicate that the small–seed phenotype is controlled by a single recessive locus, designated *ssm*.

### 2.2. BSA–Seq Identified a Candidate Interval on Chromosome 4

Resequencing DL5, T31, and the two bulks generated 638.20 Gb of clean data. The clean read rate exceeded 99% for all samples. GC content ranged from 36.4% to 36.8%, and Q30 values were ≥86.6%, indicating high sequencing quality. Reads aligned well to the SL4.0 reference genome, with mapping rates of ≥99.47%, mean sequencing depths of 175–195×, and approximately 99% genome coverage at 10× depth. Bulk construction criteria (20 individuals per bulk selected from the extremes of the seed–size distribution) and sequencing quality metrics are detailed in Section 4.5 and Supplementary Tables S2 and S3.

**Figure 3:**
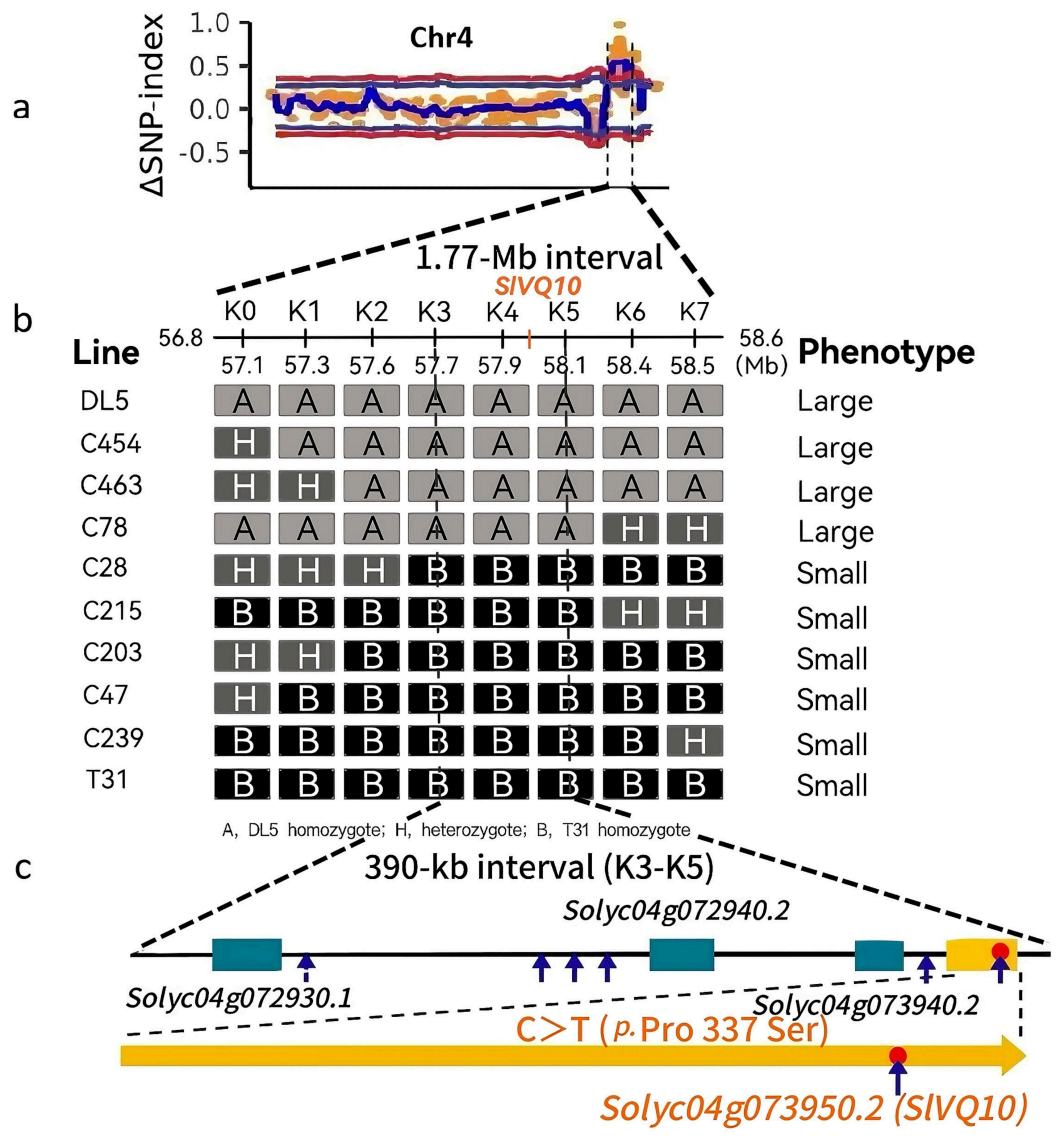
Mapping of the *ssm* locus and candidate gene identification. (**a**) ΔSNP–index plot from BSA–seq showing a significant peak on chromosome 4 corresponding to a 1.77 –Mb candidate interval. (**b**) Fine mapping with KASP markers narrowed *ssm* to a 390 –kb interval between markers K3 and K5. (**c**) EMS–type SNPs within the interval; the exonic SNP in *Solyc04g073950.2* is highlighted. Blue arrows indicate the positions of EMS–induced SNPs; teal and orange boxes represent all candidate genes contained within the interval; the orange box and red dot mark the candidate causal SNP within *Solyc04g073950.2* (*SlVQ10*).

Analysis of ΔSNP–index values revealed a single strong peak on chromosome 4, corresponding to a 1.77 –Mb candidate interval (Figure 3a). This result suggested that *ssm* is located within this region.

### 2.3. Fine Mapping Narrowed Ssm to a 390 –kb Region

KASP genotyping with eight SNP–derived markers further delimited the *ssm* locus to a 390 –kb interval between markers K3 and K5 (Figure 3b). Within this interval, six EMS–type transitions were identified. Five were intergenic and located more than 2 kb from annotated genes. Only one SNP, a C>T transition, occurred in an exon of *Solyc04g073950.2* (Figure 3c). Because it was the only non–synonymous exonic EMS–type mutation in the interval, this SNP strongly prioritized *Solyc04g073950.2* as the candidate gene.

### 2.4. Candidate Mutation and Predicted Protein Features of SlVQ10

**Figure 4:**
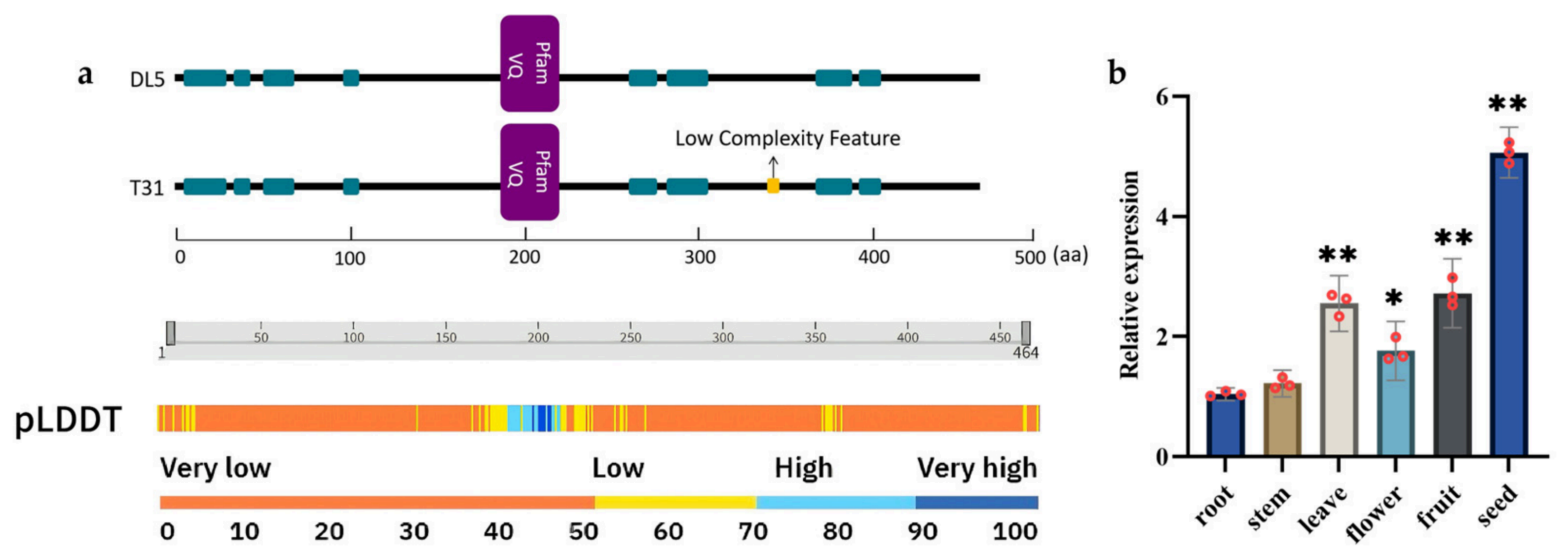
Mutation and predicted protein features of *SlVQ10* (*Solyc04g073950.2*). (**a**) The EMS–induced C>T substitution in T31 causes a Pro337Ser change and introduces a low–complexity SSGGGSS motif in the C–terminal region. The conserved VQ motif is indicated. (**b**) Tissue–specific expression pattern analysis of *SlVQ10*. Data are presented as mean ± standard deviation (*n* = 3); *, *p* < 0.05; **, *p* < 0.01; Statistical significance was assessed by two–tailed Student’s *t*–test (*p* < 0.05).

Sanger sequencing confirmed the C>T substitution in T31 at the 1011th nucleotide of the coding sequence, corresponding to Chr04:58,342,167 in the SL4.0 reference genome. This mutation caused a Pro337Ser amino acid substitution and created a low–complexity SSGGGSS motif in the C–terminal region of the protein. AlphaFold structure prediction further indicated that the region spanning the VQ motif corresponds to a segment of higher predicted confidence (pLDDT), whereas the C–terminal region containing Pro337 falls within a region of low predicted confidence, consistent with intrinsic disorder rather than a defined fold (Figure 4a).

Domain analysis showed that *Solyc04g073950.2* encodes a VQ motif–containing protein, with the conserved VQ motif located at amino acids 185–212. The mutated residue lies outside the canonical VQ domain.

### 2.5. SlVQ10 Shows Preferential Expression in Seed Tissue

qRT–PCR analysis of *SlVQ10* transcript abundance across root, stem, leaf, flower, fruit, and seed tissues of DL5 showed that *SlVQ10* was expressed in all tissues examined, with the highest relative expression detected in seed, followed by fruit and leaf; expression in stem did not differ significantly from root (Figure 4b). This tissue expression pattern, with preferential enrichment in seed, is consistent with a role for *SlVQ10* in seed development.

### 2.6. CRISPR/Cas9 Knockout Validated SlVQ10 as a Positive Regulator of Seed Size

**Figure 5:**
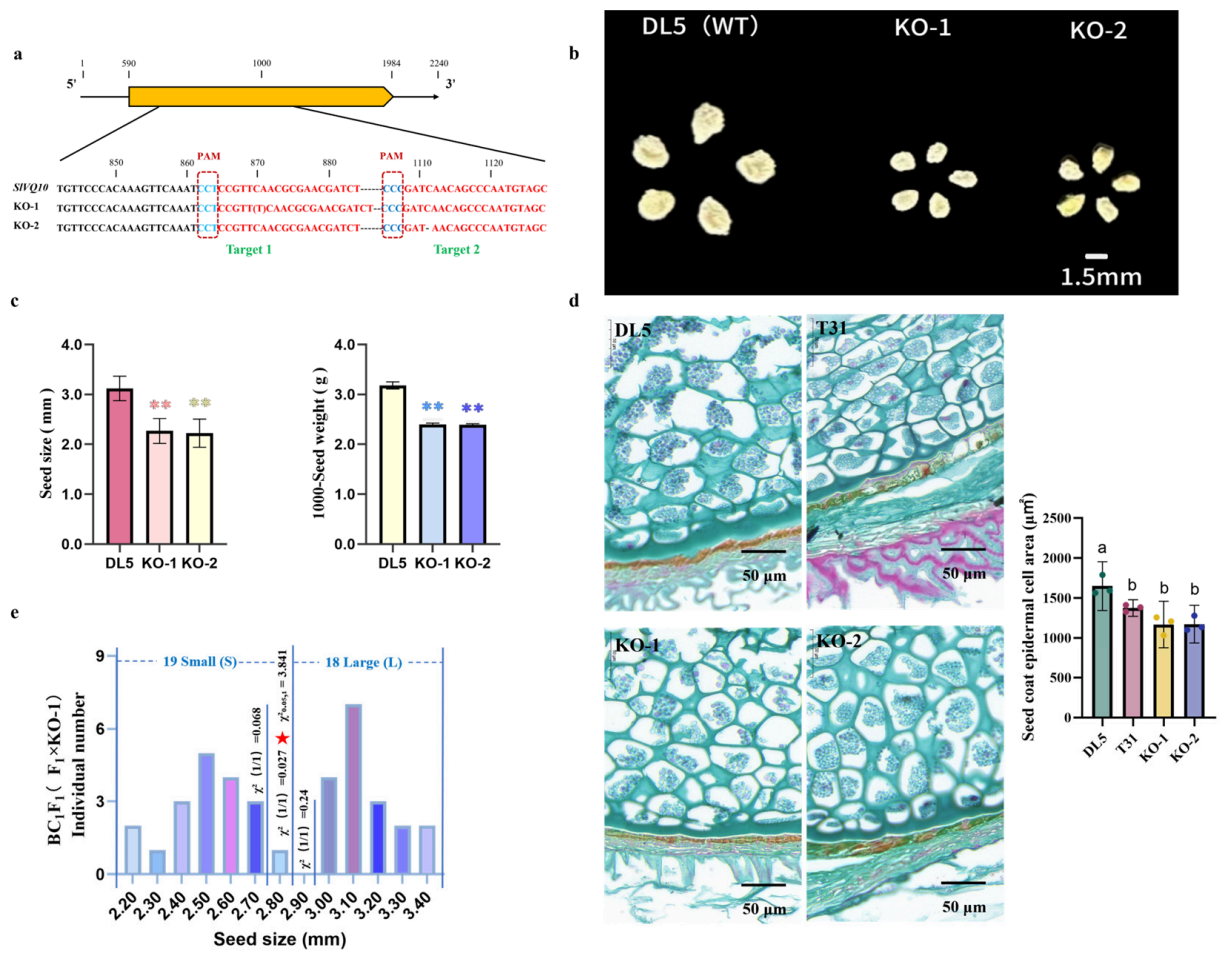
Functional validation of KO–*SlVQ10*. (**a**) CRISPR target sites and sequence confirmation of frameshift alleles in KO–1 and KO–2; Orange irregular pentagon areas represent exon. (**b**) Representative seeds from DL5, KO–1, and KO–2. (**c**) Quantification of seed size and thousand–seed weight in DL5 and knockout lines, based on measurements of at least 150 seeds per line. (**d**) Paraffin sections showing reduced seed–coat cell expansion in T31, KO–1, and KO–2 relative to DL5. Scale bar = 50 μm; Seed–coat epidermal cell areas of different lines, measured from paraffin sections using Fiji (Fiji is just ImageJ)–win64. (**e**) Segregation of seed phenotypes in BC_1_F_1_ seeds (KO–1 × F_1_), supporting co–segregation of the edited locus and the trait; Vertical lines mark the three candidate classification thresholds tested; the threshold ultimately adopted (2.80 mm, red star) gave the best fit to the expected 1:1 ratio (χ^2^ = 0.027) among the three candidates (χ^2^ = 0.068 and 0.24 for the alternative thresholds). Data are presented as mean ± standard deviation (*n* = 3); Different letters indicate significant differences among all experimental conditions. **, *p* < 0.01; Statistical significance was assessed by one–way ANOVA with Tukey’s multiple comparison test or two–tailed Student’s *t*–test (*p* < 0.05).

To test the candidate gene directly, we generated CRISPR/Cas9 knockout lines in the DL5 background. Two independent homozygous edited lines were recovered. KO–1 carried a 1 –bp insertion, whereas KO–2 carried a 1 –bp deletion. Both mutations caused frameshifts and premature translation termination (Figure 5a). The CRISPR target sites are located downstream of the region encoding the conserved VQ motif and lie within the region encoding the C–terminal portion of the protein that also contains the EMS–induced Pro337Ser substitution. The resulting frameshifts are therefore predicted to yield truncated proteins that retain an intact VQ motif but lack most of the native C–terminal sequence. PCR and Sanger sequencing of the predicted off–target sites for both sgRNAs confirmed the absence of detectable off–target edits in KO–1 and KO–2, and both lines were confirmed transgene–free by PCR screening for the Cas9/hygromycin–resistance cassette, ruling out off–target effects or residual transgene insertion as confounding explanations for the small–seed phenotype.

Both knockout lines produced significantly smaller seeds than DL5 (Figure 5b). Seed size decreased by 31.4% in KO–1 and 33.1% in KO–2. Thousand–seed weight decreased by 32.9% and 34.7%, respectively. For phenotypic description, at least 150 seeds were measured for each line; statistical significance was assessed from biological replicates (*p* < 0.01; Figure 5c). Histological observations also showed reduced seed–coat cell expansion in KO–1 and KO–2, consistent with the phenotype of T31. Quantification of seed–coat epidermal cell area showed mean values of 1648.33 ± 122.74 μm^2^ in DL5, 1374.33 ± 41.68 μm^2^ in T31 (16.6% reduction), 1165.33 ± 118.15 μm^2^ in KO–1 (29.3% reduction), and 1171.67 ± 94.50 μm^2^ in KO–2 (28.9% reduction) (Figure 5d). One–way ANOVA followed by Tukey’s multiple comparison test indicated that T31, KO–1, and KO–2 all differed significantly from DL5 (*p* < 0.05), while the three mutant/knockout genotypes did not differ significantly from one another.

No significant differences were detected between the knockout lines and DL5 for the agronomic traits measured here, including plant height, stem diameter, root length, leaf–shape index, flowering time, and fruit weight, under the tested conditions (*p* > 0.05; Supplementary Table S4). As with the F_2_ population, seed–size classification in the BC_1_F_1_ population was evaluated at the same three candidate thresholds (2.70, 2.80, and 2.90 mm). The goodness of fit to the expected 1:1 segregation ratio was assessed at each threshold (χ^2^ = 0.068, 0.027, and 0.24, respectively), and 2.80 mm, which gave the best fit, was adopted as the classification threshold for this population. Using this threshold, large and small seeds in the BC_1_F_1_ population (KO–1 × F_1_) segregated at a ratio of 52:48 (χ^2^ = 0.027, *p* = 0.87), confirming co–segregation of the edited locus with the seed phenotype (Figure 5e). The BC_1_F_1_ co–segregation test (Figure 5e) was based on 37 individuals; while the segregation ratio fit the expected 1:1 pattern (χ^2^ = 0.027, *p* = 0.87), we acknowledge that this sample size is modest for definitively excluding background effect contributions, given that the knockout lines were generated in the DL5 background and crossed to an F_1_. A larger BC_1_F_1_ population would further strengthen this conclusion.

## 3. Discussion

This study identified *SlVQ10* as the causal gene underlying a recessive small–seed phenotype in tomato. The conclusion is supported by several independent lines of evidence, including segregation analysis, BSA–seq, fine mapping, candidate mutation identification, and CRISPR/Cas9 validation. In tomato, many trait–associated genes are first suggested by expression patterns or broad genomic correlations. However, the present study followed a more direct route from mutant phenotype to causal gene. This approach provides stronger genetic support for the conclusion and makes the biological interpretation more convincing. Similar mapping–to–validation strategies have been effective in crop gene cloning and remain especially valuable for complex developmental traits [25,26,27,28,29].

This study also places a tomato VQ protein within the regulatory network of seed development. VQ proteins are widely recognized as plant–specific cofactors. They often interact with WRKY transcription factors and signaling components to regulate growth, stress responses, and transcriptional outputs [14,15,16]. In tomato, the VQ family has been identified at the genome–wide level, but its developmental functions remain poorly characterized [23]. Recent evidence that SlVQ15 can interact with SlWRKY31 in tomato supports the view that VQ–WRKY modules are active in this species, which are not limited to Arabidopsis–based models [24]. Against this background, the identification of *SlVQ10* suggests that VQ–mediated regulation in tomato contributes directly to seed growth control. This inference is further supported by the tissue expression pattern of *SlVQ10*, which showed preferential enrichment in seeds relative to vegetative tissues (Figure 4b), consistent with a direct role in seed development rather than a general growth–related function.

Histological analysis indicates that the reduction in seed size is associated with reduced seed–coat cell expansion. This observation fits well with current views of seed–size determination. Seed growth is now understood as the product of coordinated interactions among embryo growth, endosperm proliferation, the timing of cellularization, maternal tissue properties, and hormonal crosstalk [7,30,31]. Recent studies have shown that cellular dynamics during the coenocytic endosperm stage are tightly linked to final seed size. Maternal–specific regulatory pathways can alter endosperm cellularization. Endosperm turgor can also promote or constrain seed growth, depending on the developmental context [32,33,34]. In addition, seed–coat–derived brassinosteroid signaling has been shown to influence endosperm development. This further emphasizes that maternal tissues are active participants in seed–size regulation [35,36]. Yet, our study does not resolve whether *SlVQ10* acts mainly in the seed–coat, through endosperm–associated signaling, or through cross tissue coordination. Even so, the cell–level phenotype is consistent with an integrated developmental model rather than a purely embryo–autonomous one.

The EMS mutation in SlVQ10 causes a Pro337Ser substitution outside the conserved VQ core motif but introduces a low–complexity sequence in the C–terminal region. The biochemical consequence of this change is still unknown. The EMS mutant T31 showed a smaller reduction in seed size (24%) than the two independent CRISPR knockout lines (31.4–33.1%), and thousand–seed weight showed the same pattern (T31: 30.1%; KO–1/KO–2: 32.9–34.7%). This quantitative difference is consistent with T31 carrying a hypomorphic missense allele (Pro337Ser, outside the canonical VQ motif) rather than a complete loss–of–function allele, whereas the CRISPR alleles introduce frameshifts and premature stop codons that are expected to more severely disrupt protein function. We therefore interpret the EMS allele as a partial loss–of–function (hypomorphic) allele rather than a null allele; this interpretation is consistent with, rather than contradicted by, the direction and relative magnitude of the two phenotypes. Consistent with this interpretation, the CRISPR target sites lie downstream of the VQ motif, such that KO–1 and KO–2 are predicted to retain an intact VQ domain but lose most of the native C–terminal sequence, including the region altered by the EMS substitution. The more severe phenotype of these truncation alleles relative to the EMS missense allele therefore indicates that the C–terminal region beyond the VQ motif, rather than the VQ motif itself, is required for full SlVQ10 activity, and that even a local sequence change within this region (Pro337Ser) is sufficient to partially compromise this function. Furthermore, AlphaFold structure prediction indicates that this C–terminal region, including the site of the Pro337Ser substitution, is predicted with low confidence and is likely intrinsically disordered (Figure 4a); such disordered regions frequently mediate protein–protein interactions or post–translational regulation through short linear motifs, raising the possibility that the low–complexity sequence introduced by the EMS mutation per–turbs one such motif rather than a stably folded interaction surface. This interpretation is biologically reasonable, although it still requires biochemical confirmation. Given what is already known about VQ proteins, regions outside the canonical VQ motif may still contribute to partner selectivity, protein stability, or regulatory output [14,16,24].

*SlVQ10* is noteworthy because its disruption affected seed traits without causing obvious changes in several major agronomic characters measured in this study. This point matters for breeding. Developmental regulators often show undesirable pleiotropic effects. Such effects can limit their practical value. By contrast, *SlVQ10* appears to alter seed size and thousand–seed weight while leaving plant architecture, flowering time, and fruit weight largely unchanged under the tested conditions. This does not prove that the gene is universally free of trade–offs. However, it does suggest that the locus is promising enough to justify further testing in elite germplasm. This potential is reinforced by the availability of improved tomato genome resources and increasingly mature gene–editing systems, which together make translational use of validated loci more realistic than before [11,37].

Tomato breeding has historically focused on fruit–related traits, disease resistance, and shelf life. Seed traits have received less attention, even though they affect seed production efficiency and seedling performance. We believe this is one reason the present work is worth reporting. It highlights a trait category that is biologically important but often overlooked. The linked markers developed here may help breeders track the *SlVQ10* locus in segregating populations. Because the recessive *ssm* allele reduces seed size, the wild–type *SlVQ10* allele could serve as a positive marker for maintaining large seed size in breeding populations, while the linked KASP markers developed here could be used to avoid unintentional introgression of the small seed allele during backcrossing or marker–assisted selection. At the same time, the confirmed gene provides a target for genome editing.

This study still has several limitations at the mechanistic level. First, the BC_1_F_1_ co–segregation test (Figure 5e) was based on a relatively modest sample of 37 individuals; although the observed segregation fit the expected 1:1 ratio (χ^2^ = 0.027, *p* = 0.87), we acknowledge that a larger population would provide a more robust test of co–segregation, particularly given that the knockout lines were generated in the DL5 background and crossed to an F_1_. We consider this an acceptable limitation given the consistency of this result with the independent BSA–seq, fine–mapping, and phenotypic evidence supporting *SlVQ10* as the causal gene. Second, it remains unclear whether SlVQ10 interacts with WRKY proteins or other regulatory factors during tomato seed development. Third, transcriptomic and proteomic evidence is still lacking. Such evidence would help identify downstream targets and distinguish direct effects from indirect ones. Fourth, our phenotypic analysis mainly focused on seed size, TSW, and several general agronomic traits. We did not evaluate germination rate, seed vigor under stress conditions, seed composition, or endosperm–related parameters. These traits are important for assessing the practical value of *SlVQ10* in breeding. Fifth, this study did not include reciprocal crosses; therefore, we cannot formally rule out a parent–of–origin (maternal or paternal) effect on the seed–size phenotype, although the consistent recessive segregation pattern across F_2_, BC_1_, and BC_2_ populations argues against a strong parental effect. Sixth, although the convergence of two independent CRISPR/Cas9 knockout alleles, KASP–based fine mapping, and the EMS–induced missense allele provides reasonably strong genetic support for *SlVQ10* as the causal gene, we did not perform a formal complementation (rescue) experiment in which the wild–type *SlVQ10* coding sequence is reintroduced into the mutant or knockout background to restore the wild–type seed–size phenotype. Such an experiment would provide the most direct and definitive confirmation of gene identity and remains an important goal for future work. Finally, the current evidence was obtained from a single tomato genetic background. Validation in additional elite lines would further strengthen the translational relevance of this gene.

Even with these limitations, the study contributes something concrete. It identifies a causal gene rather than only a QTL. It places a tomato VQ protein in the seed–size regulatory landscape. It links gene function to a visible cellular phenotype. And it offers breeding tools that can be used immediately for further testing. This study is not just a gene–mapping exercise. It also provides a useful entry point for studying how maternal tissue growth and regulatory protein networks shape seed development in tomato.

Future work should focus on the mechanism of *SlVQ10* action. Protein–interaction assays could identify WRKY or other partners. Expression profiling during seed development could determine when and where the gene acts most strongly. Cytological analysis of endosperm and embryo development would help clarify which tissue is affected first. It would also be valuable to test whether the C–terminal low–complexity region influences protein localization, turnover, or transcriptional regulatory capacity. These next steps should make it possible to move from locus identification to pathway–level understanding.

## 4. Materials and Methods

### 4.1. Plant Materials

The small–seed mutant T31 was isolated from an EMS–mutagenized (1% *v*/*v*, 10 h) M_2_ population derived from the tomato inbred line DL5. DL5 is a homozygous red–fruited line with an average fruit weight of approximately 180 g, indeterminate growth habit, and resistance to leaf mold and tobacco mosaic virus. Plants were grown in the greenhouse of Northeast Agricultural University, Harbin, China, under a 16 h light/8 h dark photoperiod at 25 ± 2 °C and 60–70% relative humidity.

### 4.2. Phenotyping and Genetic Analysis

Seeds were collected from mature fruits on the third to fifth trusses. Because tomato seeds are oblate, seed width was used as the principal index of seed size. For each plant, 20 seeds were aligned and measured together with a digital vernier caliper. This procedure was repeated five times. The mean of the six plants within each replicate was used as the value for that biological replicate (*n* = 3 per genotype in Figure 2, Figure 4 and Figure 5). Mean seed width was then calculated from 100 seeds per plant. Thousand–seed weight (TSW) was also determined for each genotype. TSW was determined using the same replicate structure.

For inheritance analysis, six populations were generated: P_1_ (DL5), P_2_ (T31), F_1_, F_2_, BC_1_, and BC_2_. BC_1_ was generated by backcrossing F_1_ to the recurrent wild–type parent DL5, and BC_2_ was generated by backcrossing F_1_ to the mutant parent T31. Segregation was analyzed in 487 F_2_ individuals, 50 plants each of P_1_, P_2_, and BC_1_, and 144 BC_2_ plants. Chi–square tests were used to evaluate the fit to expected Mendelian segregation ratios.

For all genotypes and generations, biological replicates (individual plants) rather than individual seeds were treated as the statistical unit. Statistical comparisons among three or more groups were performed by one–way ANOVA followed by Tukey’s multiple comparison test; pairwise comparisons between two groups were performed using a two–tailed Student’s *t*–test.

### 4.3. Histological Analysis

Seeds were fixed in fresh FAA solution (90 mL 70% ethanol, 5 mL formalin, and 5 mL acetic acid), embedded in paraffin, and sectioned at 10 μm thickness. Sections were stained with safranin O–fast green and observed under a light microscope. Seed–coat epidermal cell area was measured from paraffin sections using Fiji (Fiji is just ImageJ)–win64; the two histological analyses (Figure 1 and Figure 5) were conducted independently in different batches. Statistical analysis was performed as described in Section 4.2.

### 4.4. qRT–PCR Analysis

Total RNA was extracted from the roots, stems, leaves, flowers, fruits, and seeds of the DL5 line using the TRIzol method. The RNA was then reverse–transcribed into cDNA using the HiScript III RT SuperMix kit (Vazyme, R323–01, Nanjing, China). Quantitative real–time PCR (qRT–PCR) was performed with the ChamQ Universal SYBR qPCR Master Mix kit (Vazyme, Q711, China) on a StepOnePlus Real–Time PCR System (Applied Biosystems, Waltham, MA, USA). Gene expression levels were calculated using the 2^–ΔΔCt^ method, and the samples were normalized using *SlACTIN1* (*Solyc11g005330*) and *SlACTIN2* (*Solyc03g078400*) as reference genes.

### 4.5. BSA–Seq

Twenty F_2_ individuals with the largest seeds and 20 with the smallest seeds were selected to construct the big–seed bulk (F_2_–B) and small–seed bulk (F_2_–S), respectively. Genomic DNA was extracted using the CTAB method, and equal amounts of DNA from each selected plant were pooled. The parental lines DL5 and T31 were also resequenced. Sequencing reads were aligned to the tomato reference genome SL4.0. Homozygous SNPs differentiating the parents were identified, and SNP–index and ΔSNP–index values were calculated. Candidate intervals were defined where the fitted ΔSNP–index exceeded the 95% and 99% confidence thresholds.

### 4.6. KASP Fine Mapping and Candidate SNP Analysis

Eight KASP markers were developed within the candidate interval and used to genotype 493 individuals, including 487 F_2_ plants and two plants each of F_1_, DL5, and T31. KASP assays were performed according to the LGC Genomics protocol. Because EMS predominantly induces G>A and C>T transitions, these mutation types were prioritized during candidate SNP screening.

### 4.7. Candidate Gene Sequencing and Domain Prediction

To validate the candidate mutation, *Solyc04g073950.2* was amplified from DL5 and T31 by PCR and sequenced by Sanger sequencing. Protein domain analysis was performed using SMART (https://smart.embl.de/smart/change_mode.cgi).

### 4.8. CRISPR/Cas9 Vector Construction and Phenotyping

Two sgRNAs targeting exonic regions of *Solyc04g073950.2* were designed using CRISPOR [28]. The sgRNAs were inserted into the pCAMBIA1301–Cas9 vector under the control of the SlU6 promoter [38]. The construct was transformed into DL5 through Agrobacterium–mediated cotyledon transformation. Hygromycin–resistant plants were screened, and edited alleles were confirmed by Sanger sequencing. Potential off–target sites for the two sgRNAs were predicted using CRISPOR and verified by PCR amplification and Sanger sequencing in KO–1 and KO–2; no off–target mutations were detected at any predicted site. Both knockout lines were further confirmed to be transgene–free by PCR screening for the absence of the Cas9 and hygromycin resistance cassette.

Seed traits were evaluated in two independent homozygous knockout lines (KO–1 and KO–2), with at least 150 seeds measured for each line; statistical analysis was performed as described in Section 4.2.

## 5. Conclusions

In summary, this study identifies *SlVQ10* (*Solyc04g073950.2*), a VQ motif–containing protein gene, as the causal locus underlying a stable recessive small–seed phenotype in tomato. Through a forward–genetics approach integrating BSA–seq, KASP–based fine mapping, candidate mutation analysis, and CRISPR/Cas9 validation, we provide direct genetic evidence that SlVQ10 promotes seed size in tomato. Loss of function, whether through an EMS–induced missense allele or CRISPR/Cas9–mediated knockout, reduces seed width and thousand–seed weight by approximately 24% in the EMS mutant and 31–33% in CRISPR knockout lines, and is associated with impaired seed–coat cell expansion. SlVQ10 disruption does not appear to alter major vegetative and fruit–related agronomic traits under the tested conditions, which may be advantageous for breeding applications. This work broadens our understanding of VQ protein function in crop species, showing that SlVQ10 promotes seed size and is associated with seed–coat cell expansion in tomato; whether it acts directly in the seed–coat or through cross–tissue signaling remains to be determined. The linked molecular markers and functionally validated genes provide practical resources for tomato improvement programs. Future studies should address the mechanistic basis of SlVQ10 action, including its potential interaction partners, downstream transcriptional targets, and tissue–specific expression dynamics during seed development.

## Data Availability

Data are contained within the article and Supplementary Materials. The genomic coordinates of the EMS–induced substitution in T31 (Chr04:58,342,167, SL4.0 reference genome) and the complete coding sequences of *SlVQ10* (*Solyc04g073950.2*) from both DL5 and T31 are provided in Supplementary Table S5. Because T31 carries a single, precisely mapped point substitution within the previously annotated and publicly archived *Solyc04g073950.2* locus (Sol Genomics Network/NCBI), rather than a novel or previously undescribed gene sequence, we consider that reporting the exact genomic coordinate and full CDS alignment in Supplementary Table S5 provides complete reproducibility and traceability without requiring a separate GenBank accession for a single–nucleotide variant of an already–deposited reference gene.

## Supplementary Materials

The following supporting information can be downloaded at: https://www.mdpi.com/article/10.3390/plants15152363/s1, Table S1: KASP markers used for fine mapping; Table S2: Summary of sequencing quality-control statistics; Table S3: Summary of read-mapping statistics; Table S4: Agronomic traits of DL5 and KO-*SlVQ10*; Table S5: The CDS of *SlVQ10* in DL5 and T31 lines.
