# Supplementary material for: Molecular Mapping and CRISPR/Cas9 Validation Reveal That SlVQ10 Regulates Seed Size in Tomato
Source: Plants (Basel). 2026 Jul 31;15(15):2363. doi: 10.3390/plants15152363 (PMC13467947; doi:10.3390/plants15152363)
Supplement: Supplementary file 1 [file plants-15-02363-s001.zip › Supplementary Table S1.pdf]

Molecular Mapping and CRISPR/Cas9 Validation Reveal that *SlVQ10* Regulates Seed Size in Tomato

Plants

Supplementary Table S1. KASP markers used for fine mapping

Marker ID	X allele	Y allele	Sequence [SNP]	Physical position on Chr. 4 (bp)
Soly-K0	C	T	CTTATCTTATCATTT[C/T]CTTAATAACATAA	57,183,898
Soly-K1	A	G	AAATTTGGAGAATTA[A/G]AAAAGAACATTTAT	57,338,823
Soly-K2	G	A	GACGCAAAAGAAATG[G/A]ACTACCGAAGTTTA	57,672,178
Soly-K3	C	T	CGGATACTTCATATG[C/T]CTATTTCTACATAT	57,782,509
Soly-K4	C	T	ATTTTCAACACAAAG[C/T]CTTCAGGAGGCGGA	57,924,883
Soly-K5	C	T	TCTTCCAGTGGCGGG[C/T]CAGGGCACTTACGA	58,170,496
Soly-K6	A	G	TCGTCGATTTCATCC[A/G]AATCTCTTAGCCCT	58,413,319
Soly-K7	A	G	TGTTTTGACAGCTGC[A/G]ACTTCTTTTGGTTA	58,628,227

Note. Marker positions are based on the tomato reference genome SL4.0.
