# Supplementary material for: Molecular Mapping and CRISPR/Cas9 Validation Reveal That SlVQ10 Regulates Seed Size in Tomato
Source: Plants (Basel). 2026 Jul 31;15(15):2363. doi: 10.3390/plants15152363 (PMC13467947; doi:10.3390/plants15152363)
Supplement: Supplementary file 1 [file plants-15-02363-s001.zip › Supplementary Table S2.pdf]

Molecular Mapping and CRISPR/Cas9 Validation Reveal that *SlVQ10* Regulates Seed Size in Tomato

Plants

Supplementary Table S2. Summary of sequencing quality-control statistics

Sample	Raw reads	Clean reads	Clean reads (%)	Raw bases	Clean bases	Clean bases (%)	GC content (%)	Q20 (%)	Q30 (%)
DL5	1,118,659,066	1,117,636,378	99.91	167.80 G	166.99 G	99.52	36.58	96.02	89.10
F2-B	1,076,019,492	1,074,837,422	99.89	161.40 G	160.27 G	99.30	36.42	96.88	91.15
F2-S	1,001,237,418	999,945,672	99.87	150.19 G	148.73 G	99.03	36.82	95.05	86.61
T31	1,058,736,438	1,057,653,584	99.90	158.81 G	157.83 G	99.39	36.45	96.98	91.44

Note. F2-B, big-seed bulk; F2-S, small-seed bulk.
