# Supplementary material for: Molecular Mapping and CRISPR/Cas9 Validation Reveal That SlVQ10 Regulates Seed Size in Tomato
Source: Plants (Basel). 2026 Jul 31;15(15):2363. doi: 10.3390/plants15152363 (PMC13467947; doi:10.3390/plants15152363)
Supplement: Supplementary file 1 [file plants-15-02363-s001.zip › Supplementary Table S3.pdf]

Molecular Mapping and CRISPR/Cas9 Validation Reveal that *SlVQ10* Regulates Seed Size in Tomato

Plants

Supplementary Table S3. Summary of read-mapping statistics

Sample	Total reads	Duplicate reads	Duplicates (%)	Reads after duplicate removal	Mapped reads	Mapped rate (%)	Mean coverage	Mean mapping quality	1x coverage (%)	10x coverage (%)
DL5	1,117,636,378	87,656,346	7.84	1,029,980,032	1,024,540,056	99.47	194.59x	55.506	99.65	99.14
F2-B	1,074,837,422	77,377,014	7.20	997,460,408	995,852,961	99.84	188.50x	55.2452	99.69	99.27
F2-S	999,945,672	71,531,338	7.15	928,414,334	927,447,749	99.90	175.00x	54.6619	99.66	99.12
T31	1,057,653,584	86,773,901	8.20	970,879,683	967,416,018	99.64	183.13x	54.9834	99.49	98.84

Note. F2-B, big-seed bulk; F2-S, small-seed bulk.
