# Supplementary material for: Molecular Mapping and CRISPR/Cas9 Validation Reveal That SlVQ10 Regulates Seed Size in Tomato
Source: Plants (Basel). 2026 Jul 31;15(15):2363. doi: 10.3390/plants15152363 (PMC13467947; doi:10.3390/plants15152363)
Supplement: Supplementary file 1 [file plants-15-02363-s001.zip › Supplementary Table S4.pdf]

Molecular Mapping and CRISPR/Cas9 Validation Reveal that *SlVQ10* Regulates Seed Size in Tomato

Plants

Table S4. Agronomic traits of DL5 and *ko-slvq10*

Genotype (n = 10)	Plant height (cm)	Stem diameter (mm)	Root length (cm)	Leaf-shape index (L/W)	Flowering time (d)	Fruit weight (g)
DL5	76.10 ± 0.23	5.13 ± 0.02	18.29 ± 0.12	1.42 ± 0.01	39.00 ± 0.47	182.18 ± 0.20
KO-1	76.02 ± 0.21 ns	5.12 ± 0.05 ns	18.28 ± 0.10 ns	1.43 ± 0.01 ns	39.00 ± 0.94 ns	179.88 ± 0.25 ns
KO-2	76.05 ± 0.22 ns	5.13 ± 0.02 ns	18.30 ± 0.11 ns	1.43 ± 0.01 ns	39.12 ± 0.57 ns	182.12 ± 0.28 ns

Note. ns indicates no significant difference relative to DL5, as determined by one-way ANOVA ($P > 0.05$). The reported values are means ± SD.
