# Supplementary material for: Molecular Mapping and CRISPR/Cas9 Validation Reveal That SlVQ10 Regulates Seed Size in Tomato
Source: Plants (Basel). 2026 Jul 31;15(15):2363. doi: 10.3390/plants15152363 (PMC13467947; doi:10.3390/plants15152363)
Supplement: Supplementary file 1 [file plants-15-02363-s001.zip › Supplementary Table S5.pdf]

Molecular Mapping and CRISPR/Cas9 Validation Reveal that *SlVQ10* Regulates Seed Size in Tomato

Plants

Table S5. The CDS of *SlVQ10* in DL5 and T31 lines

Lines	CDS（5’-3’）
DL5	ATGGATTCTGGGAATAGTGGGAGTATGCAATCTTCAAGTGGTGGTGATGAAGATTATGATGATTCACGTG<br>GAGAATCTATTTCTACTTTTTTGAATAATAGTAGTAATAATAATTCACTTGCCCATTTTGGTTCAATTCCA<br>TCTCATAATAATAGTAATAATCCTTCTTTTCTTTCTCATCACCATCAACAACACAACCCGAATAATACTTT<br>CTTTGATACTGCTGCTAGTTTGTTCCCACAAAGTTCAAATCCTCCGTTCAACGCGAACGATCTCATTTGGT<br>CATCATCATCAACAACATCGTCATCTCGGGCTTTAAGATCTGATCAGAGTTTGAATTTTATCTCATCAGCT<br>CCATCATCAAATGTTACTGCTAGTGGTCAACTCTTTCATCATCCTAATCAAAACCCTTTTCCGGGTTCAAG<br>TTCGTTACAAGCAATGCAACAACCATCCATGGAACCAACGAATGTTGCACGGGCCTCAAGCTCGGCCCA<br>GCCCGATCAACAGCCCAATGTAGCGAAGAACCCGAAGAAACGCACAAGAGCCTCACGGAGAGCGCCAA<br>CCACCGTGCTAACTACAGACACGACGAATTTTCGACAAATGGTTCAGGAATTCACCGGAATCCCTACGGC<br>TCCGTTCACTGGTTCACCCTATACTCGCCGTCTTGATCTGTTTTCGACAGCTGGATCGGGGATGAGGACG<br>GGACATTTGGATTCACTTGGACCATTATACCCTTTAAGGCCATCAGCACAAAAGGTTCAAGTATCTCCAT<br>TTATGTCACAATTATCATCTCCTCCTGCATCTTCGTTGAGTTCTAGCATGATTGATGCTTTAATGCCTGGA<br>AATAACAACTCTATGGTTGGTACTACCTCGGGCTCTACCTCTACTTCAACAAATTTCCAGTTAGGTAATTC<br>AAATCATGTTGGAATACAAAAACAAGCGCAAAACTTGTTCAACATGCAGAATCAAATTCTTTCATTCAAT<br>ACTGGATCAACCATTTTCAACACAAAGCCTTCAGGAGGCGGAAGCAGTACTACGATGAATGTTCCTTCGT<br>TAGATGAACTTGGAATAAGTCATGAGCAACAAGTGAGTGCAAATCTGATCAGTGGATTTCAAGGAGGGA<br>ATAATAGTAATAATAATGTTAGTAGTCAAGGAAGAAATGATGGAAATAATCTTTCAAGATTGTGGAGAA<br>ATAGTAATAATAATCATGATGGGGGTCAAGAAAATCAACGGCTGAGATCATTCGATGGGAATAATAGTA<br>ATGCTGGAAACTACAACAAGTTAAATTCAGGTAATTCTTCAACATCAGAATTTCATCCTGAGATTAATAA<br>CGGTTTGGAAAATGTGTGTTCTACTGGTGAAGGACCAGTTTCTTCATGGACTTGTCCTGATTGA
T31	ATGGATTCTGGGAATAGTGGGAGTATGCAATCTTCAAGTGGTGGTGATGAAGATTATGATGATTCACGTG<br>GAGAATCTATTTCTACTTTTTTGAATAATAGTAGTAATAATAATTCACTTGCCCATTTTGGTTCAATTCCA<br>TCTCATAATAATAGTAATAATCCTTCTTTTCTTTCTCATCACCATCAACAACACAACCCGAATAATACTTT<br>CTTTGATACTGCTGCTAGTTTGTTCCCACAAAGTTCAAATCCTCCGTTCAACGCGAACGATCTCATTTGGT<br>CATCATCATCAACAACATCGTCATCTCGGGCTTTAAGATCTGATCAGAGTTTGAATTTTATCTCATCAGCT<br>CCATCATCAAATGTTACTGCTAGTGGTCAACTCTTTCATCATCCTAATCAAAACCCTTTTCCGGGTTCAAG<br>TTCGTTACAAGCAATGCAACAACCATCCATGGAACCAACGAATGTTGCACGGGCCTCAAGCTCGGCCCA<br>GCCCGATCAACAGCCCAATGTAGCGAAGAACCCGAAGAAACGCACAAGAGCCTCACGGAGAGCGCCAA<br>CCACCGTGCTAACTACAGACACGACGAATTTTCGACAAATGGTTCAGGAATTCACCGGAATCCCTACGGC<br>TCCGTTCACTGGTTCACCCTATACTCGCCGTCTTGATCTGTTTTCGACAGCTGGATCGGGGATGAGGACG<br>GGACATTTGGATTCACTTGGACCATTATACCCTTTAAGGCCATCAGCACAAAAGGTTCAAGTATCTCCAT<br>TTATGTCACAATTATCATCTCCTCCTGCATCTTCGTTGAGTTCTAGCATGATTGATGCTTTAATGCCTGGA<br>AATAACAACTCTATGGTTGGTACTACCTCGGGCTCTACCTCTACTTCAACAAATTTCCAGTTAGGTAATTC<br>AAATCATGTTGGAATACAAAAACAAGCGCAAAACTTGTTCAACATGCAGAATCAAATTCTTTCATTCAAT<br>ACTGGATCAACCATTTTCAACACAAAGTCTTCAGGAGGCGGAAGCAGTACTACGATGAATGTTCCTTCGT<br>TAGATGAACTTGGAATAAGTCATGAGCAACAAGTGAGTGCAAATCTGATCAGTGGATTTCAAGGAGGGA<br>ATAATAGTAATAATAATGTTAGTAGTCAAGGAAGAAATGATGGAAATAATCTTTCAAGATTGTGGAGAA<br>ATAGTAATAATAATCATGATGGGGGTCAAGAAAATCAACGGCTGAGATCATTCGATGGGAATAATAGTA<br>ATGCTGGAAACTACAACAAGTTAAATTCAGGTAATTCTTCAACATCAGAATTTCATCCTGAGATTAATAA<br>CGGTTTGGAAAATGTGTGTTCTACTGGTGAAGGACCAGTTTCTTCATGGACTTGTCCTGATTGA

Data Availability Statement: The DL5 reference sequence is available under NCBI accession (LOC101250686). The T31 allele has not been independently deposited.
